# Supporting the Regional Network for Children with Burn Injuries in a Pediatric Referral Hospital for COVID-19

**DOI:** 10.3390/healthcare9050551

**Published:** 2021-05-08

**Authors:** Gloria Pelizzo, Elettra Vestri, Giulia del Re, Claudia Filisetti, Monica Osti, Anna Camporesi, Dario Rizzo, Armando De Angelis, Elena Zoia, Paola Tommasi, Gianvincenzo Zuccotti, Valeria Calcaterra

**Affiliations:** 1 Pediatric Surgery Department, “Vittore Buzzi” Children’s Hospital, 20154 Milan, Italy; elettra.vestri@asst-fbf-sacco.it (E.V.); giulia.delre@live.com (G.d.R.); claudia.filisetti@asst-fbf-sacco.it (C.F.); ostim@hotmail.com (M.O.); 2 Department of Biomedical and Clinical Science “L. Sacco”, University of Milan, 20122 Milan, Italy; gianvincenzo.zuccotti@unimi.it; 3 Department of Surgery, Policlinico San Donato, 20097 Milan, Italy; 4 Pediatric Intensive Care Unit, “Vittore Buzzi” Children’s Hospital, 20154 Milan, Italy; anna.camporesi@asst-fbf-sacco.it (A.C.); elena.zoia@asst-fbf-sacco.it (E.Z.); 5 Outpatients Unit, “Vittore Buzzi” Children’s Hospital, 20154 Milan, Italy; dario.rizzo@asst-fbf-sacco.it; 6 Burn Unit, A.O. Niguarda Ca’ Granda Hospital, 20162 Milan, Italy; armando.deangelis@ospedaleniguarda.it; 7 Pediatric Department, “Vittore Buzzi” Children’s Hospital, 20154 Milan, Italy; paola.tommasi@asst-fbf-sacco.it (P.T.); valeria.calcaterra@unipv.it (V.C.); 8 Pediatrics and Adolescentology Unit, Department of Internal Medicine, University of Pavia, 27100 Pavia, Italy

**Keywords:** COVID-19, burns, pediatric burns, management strategies

## Abstract

Considerable reorganization of the regional network for pediatric burn treatment during the pandemic was required to cope with severe burn injuries in small children. In support of the emergency network for burns during the COVID-19 pandemic, we referred to regional indications for centralization in our hospital for all children aged less than 5 years who presented with severe burns, >15% of total body surface area (TBSA), or who necessitated admittance to the pediatric intensive care unit (PICU). A new service with a dedicated management protocol was set up to treat pediatric burns in our SARS-CoV-2 pediatric hospital during the lockdown period. A multidisciplinary burn treatment team was set up to offer compassionate and comprehensive burn care. Patient’s clinical data, burn features, treatment and follow up were recorded. A higher number of admissions was recorded from February to December 2020 compared with the same period in 2019 (52 vs. 32 admissions). Eighteen patients were admitted to the COVID-19 Service (10 M/8 F; 3.10 ± 2.6 yrs); ten children (55.5%) were hospitalized in the ward and eight in the ICU (44.5%). Fifty percent of the cases presented with lesions extending over >15% TBSA; in one case, TBSA was 35%. All patients suffered 2nd-degree burns; while five patients also had 3rd degree lesions covering more than 15% TBSA. All of the injuries occurred at home. No major secondary infections were recorded. Successful treatment was achieved in 94.4% of cases. The average length of stay was 15.2 ± 12.6 days. A proactive, carefully planned service, involving a multidisciplinary team, was created to ensure appropriate care in a pediatric hospital during the COVID-19 period, despite the effective pandemic associated challenges. Better health promotion in pediatric burn cases should also include dedicated TBSA assessment and a database of children’s burn characteristics.

## 1. Introduction

The COVID 19 pandemic has affected every phase of patient care, including emergency diagnosis and management, emergency surgery, anesthesia, and peri- and post-operative management in pediatrics [1]. The “stay at home” orders during the COVID-19 period have led to changes in daily activities; and the restrictions have disarrayed the daily routine of children and adolescents with limited interactions and outdoor play. Increased activity at home has led to increased exposure to possible burn situations, explaining the significant increase in the rate of burn injures during this time in the pediatric field [2].

Almost 50% of burn injury patients in the world are children and 25% of them are admitted to hospital with severe burns [3]. Major and minor burn injuries in children require urgent medical evaluation and appropriate, dedicated treatment to avoid the recognised potential life-threatening complications, as well as aesthetic and functional sequelae [4,5].

While a significant increase in the rate of burn injures was recorded in adults during the COVID-19 period and admissions and hospitalization were often necessary due to the severity of the lesions [1], face-to-face consultation with general pediatricians was not possible during the pandemic, and fears of contracting COVID-19 in hospitals led to a decrease in parents seeking immediate attention for their child. Considerable reorganization of the regional network for pediatric burn cases during the pandemic was necessary to cope with severe burn injuries in small children.

The aim of this study was to report a strategy for the management of children with burns and the protective measures adopted at the “Vittore Buzzi” Children’s Hospital (referral centre for COVID-19), located in Milan, in the Lombardy Region, the epicenter of Italy’s coronavirus outbreak. Children were admitted to the emergency departments of the Lombardy Network hospitals following the Regional Reference Center guidelines for burns and treating pediatric emergencies during the COVID-19 period. Prior to the pandemic, a burn care team and service did not exist. Patients with superficial burns were admitted to the Emergency Unit and cases with severe lesions were referred to the adult regional Burn Reference Center.

## 2. Materials and Methods

### 2.1. Organization of the Service

To support the emergency network for burns during the COVID-19 pandemic from February 2020 onwards, we followed regional guidelines for centralization in our children’s hospital. The Vittore Buzzi Children’s Hospital was designated as a pediatric referral hospital for COVID-19 at the onset of the pandemic, and is a hub center for all surgical and medical emergencies, including a pediatric intensive care unit (PICU) with a dedicated anesthesiology team. Children aged less than five years presenting with severe burns (total body surface area, TBSA > 15%) and/or requiring PICU were admitted according to the Regional Burn Center guidelines.

A Burn Care Team, including two senior pediatric surgeons and two nurses with expertise in pediatric burns, was created and members were trained on how to implement comprehensive preventive measures against COVID-19. This team includes staff from the Pediatric Surgery Department, the Pediatric Department, and both the Anesthesiology Unit and the Emergency Department. Pediatric plastic surgeons were also included to assure adequate training in treatment in accordance with the national guidelines for burns [6].

Starting with the triage room, each child was accompanied by a single parent or caregiver. The caregiver/parent was provided with personal protective equipment (PPE) and instructed in its use according to protocols issued by the Health Management Team.

### 2.2. Patient Care

**Figure 1:**
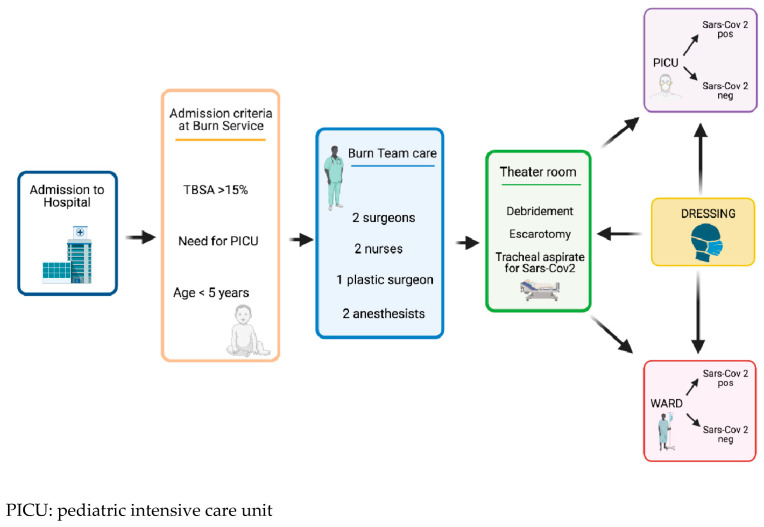
Patient care process. Created with BioRender.com (https://biorender.com/) (access on 7 May 2021).

Children were admitted together with a caregiver and both were swabbed for SARS COV-2. After admission, each patient underwent general anesthesia before debridement and escharotomy procedures and tracheal aspirate for SARS COV-2, in accordance with the anti-COVID-19 protocol. The immediate postoperative follow up was managed in a theatre room with a non-Covid pathway, used exclusively for burn patients. Patients with a confirmed diagnosis of SARS COV-2 infection and those awaiting diagnosis, were admitted to the SARS COV-2 ward or SARS COV-2–PICU areas. Patient care was provided by a multidisciplinary team which included psychologists, physiotherapists, pediatricians, nutritionists, pediatric endocrinologists, cardiologists, anesthesiologists, pediatric surgeons, infectious disease specialists, radiologists, social workers and official translators. The patient care process is summarized in Figure 1.

### 2.3. Postoperative Dressings

Collaboration with the Regional Burn Center was maintained to provide long-term follow up after discharge.

### 2.4. Patients and Data

We retrospectively examined the children admitted to the Emergency Unit of the “Vittore Buzzi” Children’s Hospital, Milan, Italy between February and December 2020. A comparison with total admissions, sex and age of the burn patients from February to December 2019 was also recorded.

At the beginning of the pandemic lockdown, inclusion criteria for admission to the Burn Service at our hospital included: age less than 5 years, severe burns (TBSA more than 15%) and/or burns requiring admittance to the PICU.

In all patients, age, sex, nationality, time of accident, admission time (time following the occurrence of the event) were recorded. At initial examination, patients underwent a SARS COV-2 swab. At admission, the burn type was determined (scald, thermal, electrical, chemical or friction), as well as the modality and etiology of injury, severity and extension of the burn lesion site (such as delicate areas like the face, hands and perineum), and possible inhalation injuries were investigated.

The site of admission (ward or PICU), the number of dressings under general anesthesia, transfusion support, social worker intervention and the length of hospital stay, were also recorded; risk of infection, antibiotic resistance and nutritional needs were monitored. Particular attention was also paid to the appearance of acute kidney injury (AKI) in its early or late presentation stages. Finally, outcomes were reviewed, including the need for follow-up.

TBSA assessment of the burn was performed according to Wallace’s ‘Rule of Nines’, which divides the body surface into areas of 9% or multiples thereof. According to the literature [5,6,7], final assessment of burns should be recorded within the first 24 h following the accident in order to accurately define the extension of the lesion.

Regarding blood loss estimates in children undergoing burn excision, for the extremities and trunk there was a 2% blood volume loss per % body surface area %BSA) excised and 5% blood volume loss per %BSA excised for the face [7]. Patients were transfused with a hemoglobin (Hb) threshold of 7 gr/dL if hemodynamically stable and with a target Hb of 10 gr/L [8,9].

### 2.5. Statistical Analysis

Data were described with the mean, standard deviation (SD), median and 25th–75th percentiles if continuous and as counts and percentages if categorical. Non-parametric correlations between continuous variables were assessed using the Spearman R test. The association of categorical variables was assessed with the Fisher’s exact test. All tests were 2-sided. A <0.05 *p*-value was considered statistically significant. Stata 16 (StataCorp, College Station, TX, USA) was used to perform the analyses.

## 3. Results

### 3.1. Epidemiological Data and Features of the Patients

A higher number of admissions was recorded from February to December 2020 compared to the same period in the previous year, 2019 (*n* = 52 vs. *n* = 32, *p* < 0.001. During the COVID-19 period, a higher prevalence of males compared to females was noted (23M/29F vs. 23M/9F, *p* = 0.01), without differences in age, Table 1.

**Figure 2:**
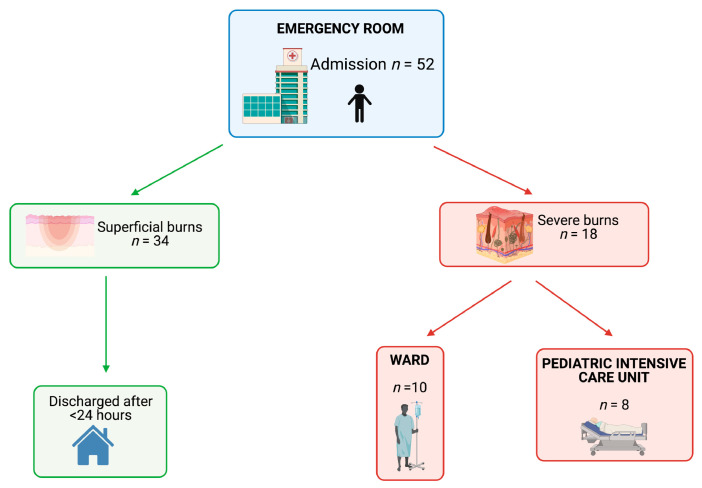
Flow chart of the children admitted to the emergency room. Created with BioRender.com (https://biorender.com/) (access on 7 May 2021).

Of the 52 subjects, 15 met the inclusion criteria for admission to the Burn Service and three other children aged over five years were also admitted to the PICU (and followed by pediatric burn team) due to the extent of their lesions (more than 20% TBSA). Of these 18 patients (10M/8F, age 3.10 ± 2.6 yrs), 10/18 (55.5%) were hospitalized in the ward and 8/18 in the ICU (44.5%). The remaining 34/52 patients (all with superficial lesions) were treated in the Emergency Room and discharged after less than eight hours of observation, Figure 2. The features of the patients are reported in Table 2.

### 3.2. Features of the Burns

The etiology and characteristics of the burns are reported in Table 3. Fifty percent of the cases (9 patients) admitted to the Burn Service had lesions extending over more than 15% TBSA (median 14%, interquartile range 2–35%), in one case the TBSA was 35%. The most common cause of burns was water. All of the patients presented with 2nd degree burns; while five patients also had 3rd degree lesions. Prior to the pandemic, all patients admitted to the Emergency Unit had superficial lesions, extending less than 10% TBSA (median 5%, interquartile range 2–8%), *p* < 0.001.

All injures occurred at home, more than 50% of them in the kitchen. Eight burn patients were admitted to the PICU and presented with a TBSA of 13% to 20%; six were aged less than five years.

The number of dressing changes performed under general anesthesia varied from 1 to 16. This number was unrelated to the type and extent of the lesion and was found to be higher in infants younger than 15 months and in patients admitted to the ward, probably due to the fact that on the ward, patients were not sedated and small children are held in their parent’s arms during the procedure.

### 3.3. Postoperative Follow-Up

In five subjects (27.7%) anemia appeared after repeated dressing (5.5 ± 0.5), requiring transfusion support (at days 18.2 ± 6.8; estimated blood loss median 200 mL, range 120–750 mL). Among these subjects, one patient (33.3%) was transfused with fresh frozen plasma + platelets + red blood cells; in four children (22.2%), under 24 months of age and with a TBSA > 15%, red blood cells only were administered.

The average length of hospital stay was 15.2 ± 12.6 days (1.3 ± 1.0 days/%TBSA). Early-stage AKI was recorded in only one patient, who was admitted to the PICU with lesions covering more than 30% TBSA; immediate medical treatment resulted in a rapid regression of the clinical evolution. In two patients (11.1%), social worker intervention was required immediately after admittance to the ward due to suspicion of child abuse in one case and drug use by the parents in the second case.

Successful burn injury treatment with closure of the wound was achieved in 18/18 (100%) of the patients. In one case, a 15-month-old patient is in follow up and is a candidate for plastic surgery to release neck scar retraction. The remaining patients were all monitored after discharge in the Outpatient Clinic, with evaluation 1–2 times per week to ensure optimum healing of the lesions and adequate physiotherapy.

### 3.4. Infection Control

At admission, only one patient showed a SARS-CoV-2-nasopharyngeal swabs positivity; the parents also were positive at admission up to discharge. No transmission between positive and negative patients for SARS-CoV-2 in the ward and PICU was recorded. After hospital discharge, no familial infections are reported.

No major wound infections following the burn treatments were noted, even in patients positive for SARS-CoV-2. Skin swabs showed three cases of Gram negative infection (burn grade 2nd and 3rd in all).

SARS-CoV-2 infection was recorded in four nurses and two surgeons at the beginning of the epidemic (14.8%). No trasmission between the patients and health care workers was recorded.

## 4. Discussion

Burn patients are usually debilitated and require multiple operative procedures, which carry an increased risk of coronavirus infection and transmission both for the patient and the caregivers. It is advisable to consider every child treated in an emergency setting as a potential COVID patient.

Thus, considerable reorganization of the Pediatric Service was carried out in order to support and protect the well-being of pediatric burn cases and to avoid the negative impact of the pandemic on the long-term consequences of pediatric burn injuries.

Mortality in pediatric burns is closely related to patient age [3,10,11] and etiology of the burn; flame burns are the most common cause of death. Mortality and morbidity are also associated with higher degree burns.

The extent of TBSA is estimated as the percentage of the total body surface area that is injured by dermal and full-thickness burns. A burn area of more than 20% is associated with a significantly higher mortality rate (two-thirds) than that of patients with a TBSA of less than 20% [12,13,14].

Moreover, morbidity is higher for children admitted to hospital more than four hours after the accident occurrs. During lockdown, a significant increase in the rate of burn injures in children was recorded, associated with delayed presentation to the Emergency Department. We noted delayed admission in 27.7% of the patients. Late admission causes a delay in fluid resuscitation, burn wound treatment, pain control, and wound infection containment, thus influencing the prognosis.

The regional network for children experiencing burns recorded 28 cases in 2019. Among them, seven were patients under the age of five years, with a TBSA of less than 15% and no need for PICU admission. In our cohort, 50% of the cases presented lesions extending over more than 15% TBSA; in one subject, TBSA was 35%. Burns exceeding 25–30% TBSA present with edema in all tissues and massive activation of a cytokine-mediated inflammatory response, which call for extremely challenging treatments in pediatrics. This condition also induces an early systemic effect, burn shock, followed later by a hypermetabolic phase [15].

In the literature, TBSA overestimation is reported in adults and children [16,17]. Burn size estimation remains a problem and is found to be overestimated by more than 5% in nearly 50% of patients. As reported in the literature, a 2–3% margin of error in TBSA may lead to clinical compromise, especially in burns involving more than 15–20% TBSA and in small children [16,17,18]. In our experience, the TBSA estimation was recorded within 24 h. In fact, early TBSA assessments are more accurate for diagnoses than those performed more than 24 h apart. This indicates that there is an advantage of treating children’s burns in a Pediatric Center that has a PICU with a dedicated pediatric team who are experts in the hemodynamic pathophysiology of children. Moreover, the teams caring for these children have been proactive in ensuring appropriate follow-up and accessible healthcare advice despite the challenges this pandemic has brought. Thus, a dedicated pediatric burn teams could be proposed in emergency conditions in a pediatric hospital as a model able to optimize the results and quality of care offered by the regional referral center for burn injures.

To optimize burn management in children, firstly, it is mandatory to accurately assess TBSA in order to arrange for the patient’s transfer to a burn center as soon as possible and to perform immediate fluid resuscitation. Secondly, a pediatric burn database is necessary in order to classify all types of pediatric lesions and to provide an opportunity to review data for specific treatments. Finally, a proactive infection control approach is suitable in burn units. In the multidisciplinary team, the inclusion of an infectious disease specialist and a pharmacist is highly recommended to optimize the quality of care [19]. Access to standard operating procedures and a description of outcomes for such a service would be useful in the optimization and re-organization of Regional Burn Networks.

## 5. Conclusions

The treatment of pediatric burn patients in a SARS COV-2 reference hospital should be considered safe and feasible, with no additional risks of infection or major sequelae. A carefully planned service, involving a multidisciplinary team to protect the well-being of patients and parents in pediatric hospitals during the Covid-19 pandemic, facilitated cooperation with the Regional Burn Center for long-term treatment and care.

## Figures and Tables

**Table 1:** Features of the patients admitted to Emergency Unit in February to December 2020 and in the same period in the previous year 2019.

Features	Year 2020	Year 2019
Number of admission, *n*	52	32
Sex (M/F)	23/29	23/9
Age	3.9 ± 4	4.0 ± 4.4
Burn grade *n*		
First	34	32
2nd	13	0
2nd and 3rd	5	0
Total burn surface (%)		
<10	32	32
10–15	11	0
>15	9	0

**Table 2:** Features of the patients admitted to the COVID-19 Service.

Features	Results
Number, *n*	18
Sex (M/F)	10/8
Age (years)	3.10 ± 2.6
Nationality, *n* (%)	
Italian	6 (33.3)
Other	12 (66.7)
Positivity SARS-CoV-2—nasopharyngeal and BAL of patient, *n* (%)	1 (5%)
Time of accident *n* (%)	
Morning	6 (33.3)
Afternoon	7 (38.9)
Night	5 (27.8)
Admission time (following the occurrence of the event), *n* (%)	
early (<4 h)	13 (72.3)
delayed (>4 h)	5 (27.7)

**Table 3:** Features of the burns.

Features	Number of Patients (%)
Burn grade *n* (%) 2nd 2nd and 3rd	13 (72.2) 5 (27.7)
Source, *n* (%)	
Water	13 (72.2)
Oil	1 (5.5)
Flame	1 (5.5)
Oven	1 (5.5)
Coal	1 (5.5)
Thermal	1 (5.5)
Total burn surface (%) <15% ≥15%	13.0 ± 7.8 9 (50) 9 (50)

## Data Availability

All data are reported in the manuscript.

## References

[1] Shekerdemian L.S., Mahmood N.R. (2020). Characteristics and outcomes of children with coronavirus disease 2019 (COVID-19) infection admitted to US and Canadian pediatric intensive care units. JAMA Pediatrics.

[2] Holland A.J. (2006). Pediatric burns: The forgotten trauma of childhood. Can. J. Surg..

[3] Sethuraman U., Stankovic C., Singer A., Vitale L., Krouse C.B., Cloutier D., Donoghue L., Klein J., Kannikeswaran N. (2021). Burn visits to a pediatric burn center during the COVID-19 pandemic and ‘Stay at home’ period. Burns.

[4] Ryan C.M., Schoenfeld D.A. (1998). Objective estimates of the probability of death from burn injuries. N. Engl. J. Med..

[5] Suman A., Owen J. (2020). Update on the management of burns in paediatrics. BJA Educ..

[6] Toon M.H., Maybauer D.M. (2011). Children with burn injuries—assessment of trauma, neglect, violence and abuse. INJ Violence Res..

[7] Ranno R., Vestita M. (2020). Italian recommendations on enzymatic debridement in burn surgery. Burns.

[8] Palmieri T.L. (2017). Children are not little adults: Blood transfusion in children with burn injury. Burns Trauma.

[9] Doctor A., Cholette J.M., Remy K.E., Argent A., Carson J.L., Valentine S.L., Bateman S.T., Lacroix J., Pediatric Critical Care Transfusion and Anemia Expertise Initiative (TAXI), Pediatric Critical Care Blood Research Network (BloodNet), and the Pediatric Acute Lung Injury and Sepsis Investigators (PALISI) Network (2018). Recommendations on RBC transfusion in general critically ill children based on hemoglobin and/or physiologic thresholds from the Pediatric Critical Care Transfusion and Anemia Expertise Initiative. Pediatr. Crit. Care Med..

[10] Tieu B.H., Holcomb J.B., Schreiber M.A. (2007). Coagulopathy: Its pathophysiology and treatment in the injured patient. World J. Surg. 2007, 31, 1055–1064. World J. Surg..

[11] Wolf S.E., Rose J.K. (1997). Mortality determinants in massive pediatric burns. An analysis of 103 children with > or = 80% TBSA burns (>or = 70% full-thickness). Ann. Surg..

[12] Bessey P.Q., Arons R.R. (2006). The vulnerabilities of age: Burns in children and older adults. Surgery.

[13] Chan Q.E., Barzi F. (2012). Burn size estimation in children: Still a problem. Emerg. Med. Australas.

[14] Bland J.M., Altman D.G. (1999). Measuring agreement in method comparison studies. Stat. Methods Med. Res..

[15] Tan Chor Lip H., Tan J.H. (2019). Survival analysis and mortality predictors of hospitalized severe burn victims in a Malaysian burns intensive care unit. Burn. Trauma.

[16] Nielson C.B., Duethman N.C. (2017). Burns: Pathophysiology of systemic complications and current management. J. Burn. Care Res..

[17] Swords D.S., Hadley E.D. (2015). Total body surface area overestimation at referring institutions in children transferred to a burn center. Am. Surg..

[18] Bittner E.A., Shank E. (2015). Acute and perioperative care of the burn-injured patient. Anesthesiol.

[19] Lachiewicz A.M., Hauck C.G., Weber D.J., Cairns B.A., van Duin D. (2017). Bacterial Infections After Burn Injuries: Impact of Multidrug Resistance. Clin. Infect. Dis..

